# High spectral response of self-driven GaN-based detectors by controlling the contact barrier height

**DOI:** 10.1038/srep16819

**Published:** 2015-11-17

**Authors:** Xiaojuan Sun, Dabing Li, Zhiming Li, Hang Song, Hong Jiang, Yiren Chen, Guoqing Miao, Zhiwei Zhang

**Affiliations:** 1 State Key Laboratory of Luminescence and Applications, Changchun Institute of Optics, Fine Mechanics and Physics, Chinese Academy of Sciences, Changchun 130033, People’s Republic of China

## Abstract

High spectral response of self-driven GaN-based ultraviolet detectors with interdigitated finger geometries were realized using interdigitated Schottky and near-ohmic contacts. Ni/GaN/Cr, Ni/GaN/Ag, and Ni/GaN/Ti/Al detectors were designed with zero bias responsivities proportional to the Schottky barrier difference between the interdigitated contacts of 0.037 A/W, 0.083 A/W, and 0.104 A/W, respectively. Voltage-dependent photocurrent was studied, showing high gain under forward bias. Differences between the electron and hole mobility model and the hole trapping model were considered to be the main photocurrent gain mechanism. These detectors operate in photoconductive mode with large photocurrent gain and depletion mode with high speed, and can extend GaN-based metal-semiconductor-metal detector applications.

The GaN-based material system is well suited to application as a photodetector material for operation in the 200–365 nm wavelength range because of its tunable wide direct bandgap. In addition, photodetectors made using this material system also have the advantages of being solid-state and small in size, with good chemical and thermal stability, thereby saving energy in operation and having long lifetimes. Potential uses of such ultraviolet (UV) photodetectors include flame monitoring and detection, vegetation growth monitoring, ozone layer monitoring, UV astronomy, gas detection, water purification, submarine communications, and medical applications^1^,^2^,^3^. These photodetectors are also chemically inert and are thus suitable for harsh environments. To date, many encouraging advances in GaN-based detectors have been reported using a variety of device structures, including Schottky-type^4^,^5^,^6^, metal-semiconductor-metal (MSM)-type^7^,^8^,^9^,^10^,^11^, p–i–n type and avalanche-type photodetectors (APDs)^12^,^13^,^14^. Among these detectors, the MSM structure is an attractive candidate for UV photodetector applications, because this type of detector has low dark current, low noise and high response speed characteristics^15^,^16^. In addition, its growth and fabrication processes are simplified because n- and p-type doped layers are not required. Normally, MSM structures can use either back-to-back Schottky contacts during photovoltaic operation, or back-to-back ohmic contacts during photoconductive operation. However, neither of these types of device can operate under 0 V bias.

The Schottky contact photodiode is best suited for applications that require fast response speeds and low dark current, while the ohmic contact photoconductor is preferred for high photosensitivity applications. It is desirable that a GaN-based interdigitated finger detector can work under 0 V bias, and it is also desirable that this type of detector can work in both photoconductive and depletion modes so that high photosensitivity and rapid response speeds can both be realized with a single device.

Up to now, self-driven GaN detectors with MSM structure has been realized by introducing Ni and Au as the interdigitated finger electrodes^17^. However, the peak responsivity of this kind of detector under 0 V is only 0.005 A/W. Furthermore, this kind of detector can not work in photoconductive mode to realize high gain. In this paper, based on the principle of Schottky and Ohmic contacts and MSM-type detectors, we propose a structure to enable tuning one of the MSM-type interdigitated finger contacts from Schottky to Ohmic. Using this structure, high responsivity of self-driven GaN-based detectors with interdigitated finger geometries were realized, which is about 0.104 A/W at 0 V bias for Ni/GaN/Ti/Al detector and the quantum efficiency is about 36%. Compared with that of the Ni/GaN/Au detector, the responsivity of the Ni/GaN/Ti/Al at 0 V bias is more than 20 times enhanced. Furthermore, this type of detector can also operate in both photoconductive and depletion modes.

## Results

**Figure 1:**
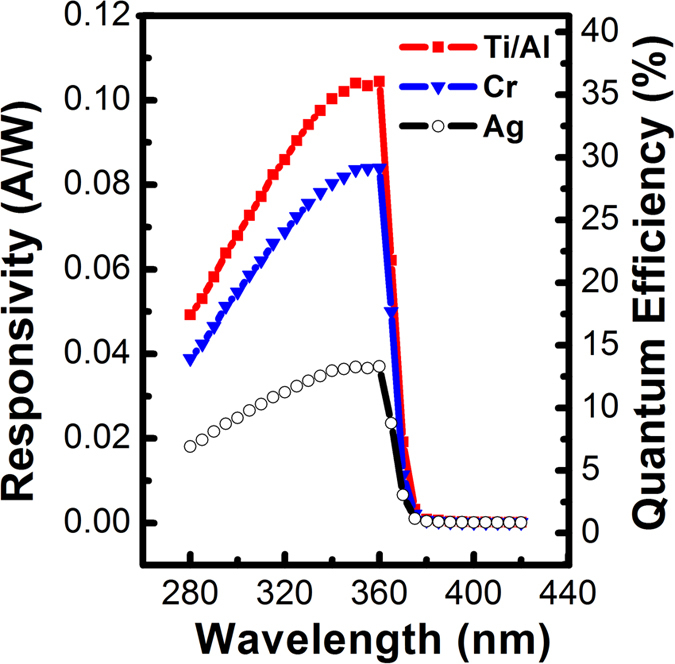
Spectral responses of Ni/GaN/Cr, Ni/GaN/Ag and Ni/GaN/Ti/Al detectors under 0 V bias.

The spectral responses of many Ni/GaN/Cr, Ni/GaN/Ag and Ni/GaN/Ti/Al structures under 0 V bias were investigated. All of these structures showed self-driven properties and the typical responsivities under 0 V bias of the Ni/GaN/Cr, Ni/GaN/Ag and Ni/GaN/Ti/Al structures, named samples A, B and C ,respectively, are shown in Fig. 1. All the three types of device had sharp cut-offs at the GaN band edge and showed high responsivities. However, the peak responsivity (R) values differed for the three device types, with results of about 0.037 A/W, 0.083 A/W and 0.104 A/W for samples A, B and C, correspondingly to the quantum efficiency of about 13%, 29% and 36%, respectively. These results followed a sequence of R_*A*_ < R_*B*_ < R_*C*_.

## Discussion

Comparison of the three detectors showed that all of them contained the same interdigitated Ni contacts, but their other interdigitated finger contacts were different. Because the metal work function (W) values for the four kinds of interdigitated contact metals have the sequence of W_*Ni*_ > W_*Cr*_ > W_*Ag*_ > W_*Ti/Al*_, the falls in the metal work function (ΔW) between the two interdigitated contacts of the three samples are consistent with the sequence ΔW_*A*_ < ΔW_*B*_ < ΔW_*C*_. Therefore, it seems that the responsivity of the self-driven GaN-based detectors is dependent on ΔW. The larger that ΔW is, then the higher that the responsivity becomes. Because the metal work function affects the Schottky barrier height directly, it is reasonable to deduce that a large value of ΔW between the interdigitated contacts results in a large fall in the Schottky barrier, and thus large band bending between the two interdigitated finger contacts. Then, the photogenerated electron-hole pairs are able to drift more easily to the electrode and be collected, leading to higher responsivity under 0 V bias. This is a photovoltaic effect of a Schottky junction, similar to the solar cell.

**Figure 2:**
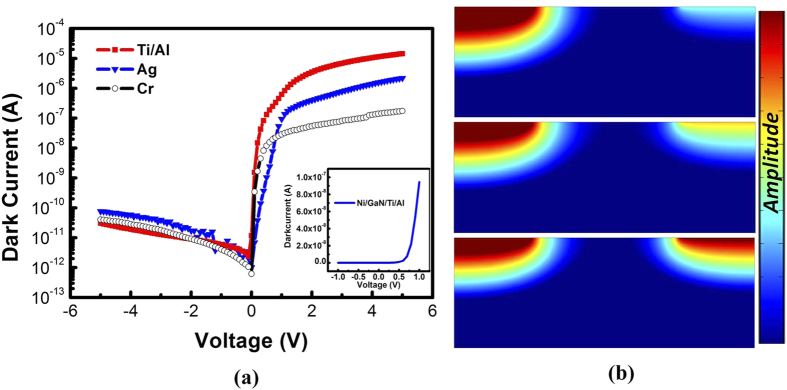
(**a**) Correlation between the dark current and the voltage for the Ni/GaN/Cr, Ni/GaN/Ag and Ni/GaN/Ti/Al detectors with the inlet of the dark current property of Ni/GaN/Ti/Al in linear scale. **(b**) Simulated spatial distributions of the electric field intensities of the Ni/GaN/Ti/Al, Ni/GaN/Ag and Ni/GaN/Cr structures.

To confirm this speculation, the dark current-voltage (*I*–*V*) curves of the three typical detectors were measured, as shown in Fig. 2(a). All measurements began at a reverse 5 V bias at the Ni/GaN contact. All *I*–*V* curves for the three samples showed asymmetrical shapes between the reverse and forward voltage regions. While the dark currents for all samples showed nearly identical typical Schottky behavior in the reverse voltage region, the *I*–*V* curves showed differences in the forward region. In this paper, the ‘forward’ bias means applying positive bias to the side with higher barrier, i.e., GaN/Ni. Under the same applied bias, the dark currents (D) of the detectors followed the sequence D_C _> D_B _> D_A_. For sample C, i.e., the Ni/GaN/Ti/Al detector, the dark current was nearly six orders of magnitude higher under the forward 5 V bias than that under the reverse 5 V bias, similar to the behavior of a GaN-based Schottky barrier photodiode.

The Schottky barrier height (SBH) of the interdigitated finger structure can be calculated approximately using the dark current at the reverse junction since the metal-semiconductor junction under reverse voltage dominates the dark current^17^. Based on the thermal thermionic emission model^18^,


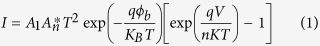


where *A*_*1*_ is the photodetector area, *A*_*n*_ is the effective Richardson constant, and 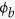 is the SBH. q is the elementary charge, V is the applied voltage, and n is the ideality factor. The SBHs for the GaN/Ni, GaN/Cr, GaN/Ag, and GaN/Ti/Al structures were approximately 0.85 eV, 0.57 eV, 0.34 eV, and 0.08 eV, respectively. It is reasonable that the SBHs decreased with decreasing contact metal work function and the GaN/Ti/Al contact was nearly ohmic. Therefore, the asymmetric I-V behavior could be explained for all three detectors as follows: in the reverse region, the Ni/GaN Schottky contact was under reverse junction bias and dominated the dark current and the SBH, resulting in the dark current in this region being similar to that of GaN-based MSM detectors with back-to-back Schottky contacts. In contrast, in the forward region, the metal with the lower work function determined the SBH, leading to a lower SBH and much higher dark currents. For the Ni/GaN/Ti/Al structure, the typical interdigitated Schottky and near-Ohmic contacts resulted in the largest difference in current between the forward and reverse regions.

**Figure 3:**
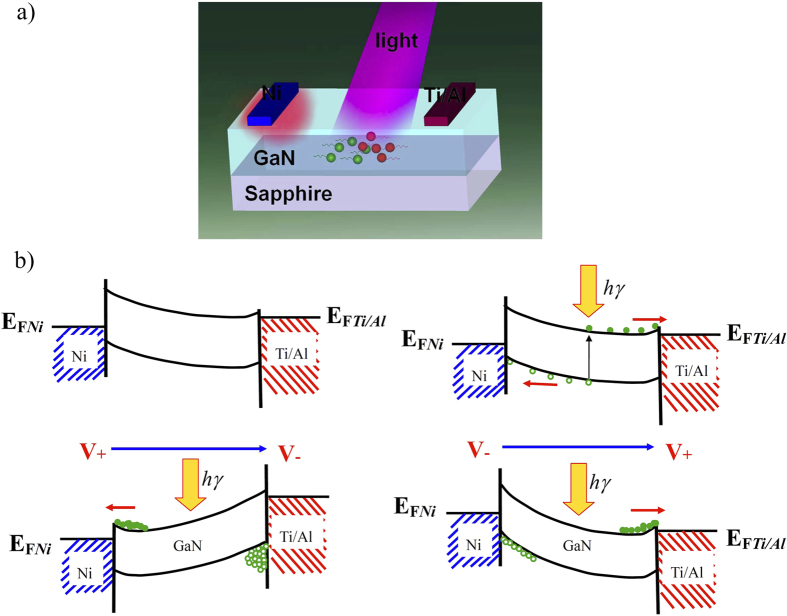
(**a**) Schematic analysis of the self-driven properties of the Ni/GaN/Ti/Al detector. **(b**) the band diagram of a Ni/GaN/Ti/Al detector before and after illumination.

The spatial distributions of the electric field intensity were also simulated using time-domain and frequency-domain finite-element methods. Figure 2(b) shows the simulation results for the Ni/GaN/Ti/Al, Ni/GaN/Ag and Ni/GaN/Cr structures. The results show that the spatial distribution of the electric field intensity depends on the SBH. The higher that the SBH is, then the stronger that the electric field intensity becomes. The spatial distribution of the electric field intensity for the Ni/GaN contact is the strongest, while that for the GaN/Ti/Al contact is the weakest. Thus, the self-driven properties of these detectors can mainly be attributed to the Schottky barrier drop, as shown by the schematic analysis in Fig. 3(a). The different interdigitated Schottky and near-ohmic contacts result in a fall in the Schottky barrier, and thus in band bending. Greater differences between the two interdigitated Schottky barriers mean that greater band bending will occur. Then, the photogenerated electron-hole pairs, especially those in the depletion region, will drift to the electrode and can be collected. As a result, the GaN-based detectors with interdigitated finger contact structures have their self-powered properties. The band diagram of the detectors before and after illumination were given in Fig. 3(b).

**Figure 4:**
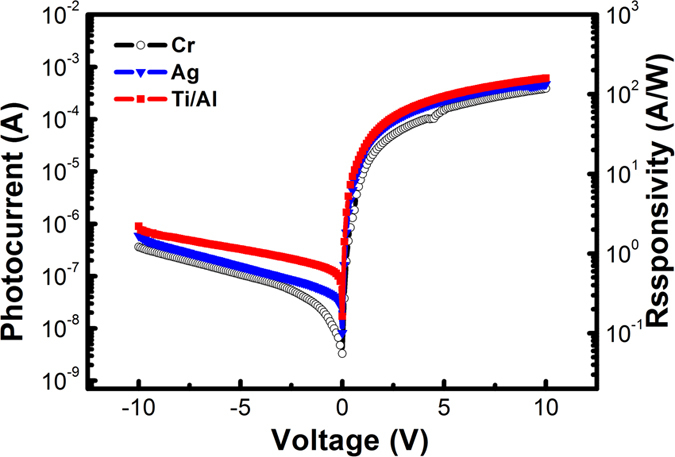
Correlation between the photocurrent and the voltage of the Ni/GaN/Cr, Ni/GaN/Ag and Ni/GaN/Ti/Al detectors.

However, the depletion layer width of the Ni/GaN structure can be calculated as follows:


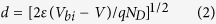


where *ε*, *V*_*bi*_, and *V* are the dielectric permittivity, the Schottky built-in voltage, and the external bias voltage, respectively, and *N*_*D*_ is the layer doping level. Therefore, for the Ni/GaN Schottky contact, the depletion layer width with zero bias is no more than 100 nm, which is much narrower than the electrode gap. Thus, in addition to the fall in the Schottky barrier, there may be some other associated reasons that contribute to the high responsivity under 0 V bias. To figure out the mechanism, the voltage-dependent photocurrent was measured upon illumination at 360 nm, as shown in Fig. 4, where the right-hand vertical axis shows the corresponding responsivity. All three devices show the existence of an internal gain in both the forward and reverse regions. One possible reason for this is the difference between the electron and hole mobilities, which is the typical photoconductive gain mechanism, and another possible reason is hole trapping^19^. In our specific cases, the near-ohmic contacts dominate the current under the applied forward bias, and thus the photoconductive gain mechanism may dominate the internal gain in the forward region. If the photoconductive gain mechanism alone exists, then there will be a linear responsivity dependence on bias, which can be understood as follows:


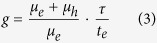



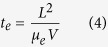


Where 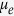 and 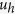 are the electron and hole mobilities, respectively. 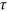 is the recombination lifetime, *t*_*e*_ is the electron transit time, *L* is the electrode gap spacing and *V* is the applied bias. Based on the above equations, an increase in *V* leads to reduction of *t*_*e*_, and thus the gain subsequently increases linearly. However, the *I*–*V* curves under illumination do not maintain a linear relationship, and we therefore speculate that hole trapping may also contribute to the high gain when we consider that the dislocation density in our GaN epilayer is approximately 10^9^ cm^2^. However, Compared with the hole concentration in GaN, the density of trapped holes is so little that the effect of the trapped holes on the hole mobility can be ignored. The hole trapping assists the high responsivity can be explained as follows: hole trapping leads to the sweep-out and reinjection of electrons to maintain charge neutrality in the space-charge region. Electron reinjection dominates the photoresponse and thus constitutes the gain mechanism. This mechanism is also suitable for the gain in the reverse region. The high responsivities of our self-driven detectors with their interdigitated geometries can thus be summarized as follows: the Schottky barrier drop between the interdigitated contacts leads to band bending, which makes the photogenerated carriers drift; then, hole trapping assists the high responsivity under 0 V bias.

**Figure 5:**
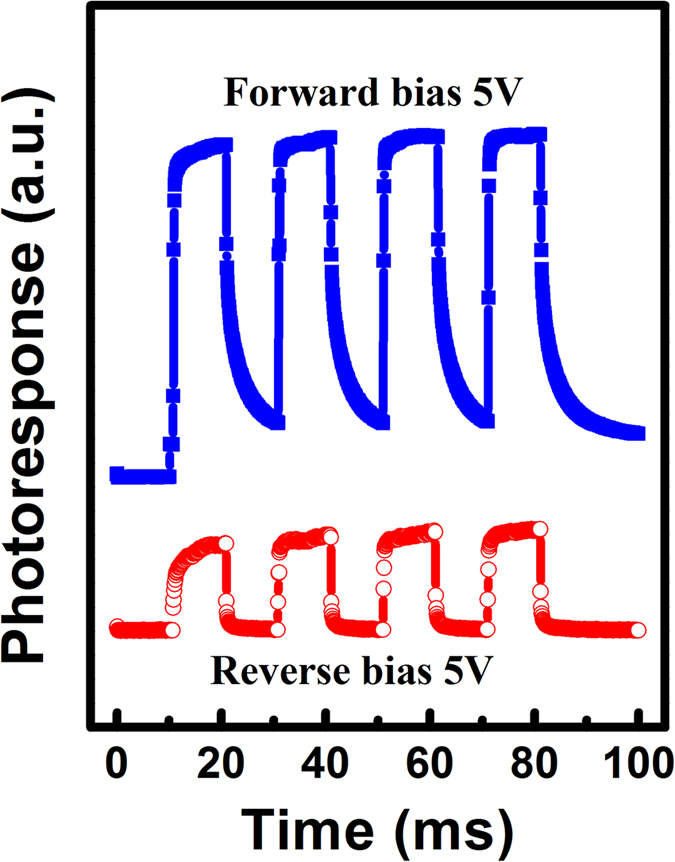
Transient responses of the Ni/GaN/Cr, Ni/GaN/Ag and Ni/GaN/Ti/Al detectors.

The transient responses of the detectors are also studied and the response times for all detectors are faster in the reverse bias mode than those in the forward bias mode. Figure 5 shows the transient response under 360 nm illumination and 5 V bias for the Ni/GaN/Ti/Al detector. The figure shows that the response time is more than 10 ms under 5 V forward bias because of the photoconductive mode. In contrast, the response time under a reverse bias of 5 V is fast because of the depletion mode Ni/GaN Schottky contact, which determines the response time under reverse bias. Therefore, it can be concluded that the self-driven GaN-based detectors with interdigitated geometries can work in both depletion mode with fast response speed and photoconductive mode. In addition, the response times shown here again confirm our analysis of the gain mechanism.

In conclusion, self-driven GaN-based detectors with interdigitated structures were realized by adopting interdigitated Schottky and near-ohmic contacts. The fall in the Schottky barrier between the interdigitated contacts caused a difference between the spatial distributions of the electric field intensity and subsequent band bending. Therefore, the photogenerated holes and electrons drifted and were collected by the electrodes. The spectral response under 0 V bias increased with increasing difference between the interdigitated Schottky barrier heights, and the responsivity of the Ni/GaN/Ti/Al detector was as high as 0.104 A/W under 0 V bias. Hole trapping also contributed to the high responsivity of the detectors. In addition, this type of detector can work in both depletion mode with fast response speed and conductive mode with high photosensitivity.

## Methods

The undoped GaN epilayer used in our devices was grown on c-plane sapphire substrates at 1050 °C by metalorganic chemical vapor deposition (MOCVD). The details of the crystal growth process have been reported previously^10^. The room temperature carrier concentration for the active GaN layer was approximately 3 × 10^16^ cm^-3^ and its dislocation density was approximately 10^9^ cm^-2^. Three typical interdigitated finger structures of Ni/GaN/Ag, Ni/GaN/Cr and Ni/GaN/Ti/Al were designed and fabricated. The interdigitated fingers were 5 μm wide and 100 μm long, with 5 μm spacing.

For the interdigitated Schottky contact, Ni (80 nm) was deposited by electron beam evaporation followed by lift-off processes. The Ni layer was subsequently annealed by rapid thermal annealing at 500 °C for 180 s. The other interdigitated contact was fabricated by a similar process. First, photolithographic alignment was performed; then, Ag (80 nm) was deposited by electron beam evaporation and a second lift-off process, followed by rapid thermal annealing at 450 °C for 180 s to complete the fabrication of the Ni/GaN/Ag structure. Similarly, Ni (80 nm)/GaN/Cr (80 nm) and Ni (80 nm)/GaN/Ti (20 nm)/Al (60 nm) structures were also completed. In these structures, Cr was annealed by the rapid thermal annealing system at 500 °C for 180 s, while the Ti/Al layer was annealed at 600 °C for 30 s.

To enable study of the quality of the Schottky barriers, an indium ohmic contact was deposited near the edge of the sample. The voltage-dependent photocurrent and the dark current-voltage (*I*–*V*) characteristics were measured; the details of these measurements can be found in^10^. The response times of the detectors were measured by switching the light on and off at the UV wavelength of 360 nm while the devices were connected to a Keithley 6487 electrometer. All measurements were carried out at room temperature.

## Additional Information

**How to cite this article**: Sun, X. *et al.* High spectral response of self-driven GaN-based detectors by controlling the contact barrier height. *Sci. Rep.* **5**, 16819; doi: 10.1038/srep16819 (2015).

## References

[b1] Razeghi M. & Rogalski A. Semiconductor ultraviolet detectors J. Appl. Phys. 79(10), 7433–7473 (1996).

[b2] Ozbay E. *et al.* High-performance solar-blind photodectors based on AlxGa1-xN heterostructures. IEEE J. Quantum Electron. 10(4), 742–751 (2004).

[b3] Pearton S. J., Zolper J. C., Shul R. J. & Ren F. GaN: Processing, defects, and devices. J. Appl. Phys. 86 **(1)**, 1–78 (1999).

[b4] Chen Q. *et al.* Schottky barrier detectors on GaN for visible–blind ultraviolet detection. Appl. Phys. Lett. 70(17), 2277–2279 (1997).

[b5] Katz O., Garber V., Meyler B., Bahir G. & Salzman J. Anisotropy in detectivity of GaN Schottky ultraviolet detectors: Comparing lateral and vertical geometry. Appl. Phys. Lett. 80(3), 347 (2002).

[b6] Lee M. L. *et al.* GaN Schottky barrier photodetectors with a low-temperature GaN cap layer. Appl. Phys. Lett. 82(17), 2913–2915 (2003).

[b7] Li J., Xu Y., Hsiang T. Y. & Donaldson W. R. Picosecond response of gallium-nitride metal–semiconductor–metal photodetectors. Appl. Phys. Lett. 84(12), 2091–2093 (2004).

[b8] Mosca M., Reverchon J. L., Omnes F. & Duboz J. Y. Effects of the buffer layers on the performance of (Al, Ga)N ultraviolet photodetectors. J. Appl. Phys. 95(8), 4367–4370 (2004).

[b9] Pau J. L. *et al.* Response of ultra-low dislocation density GaN photodetectors in the near- and vacuum-ultraviolet. J. Appl. Phys. 95(12), 8275–8279 (2004).

[b10] Li D. B. *et al.* Influence of threading dislocations on GaN-based metal-semiconductormetal ultraviolet photodetectors. Appl. Phys. Lett. 98(1), 011108 (2011).

[b11] Sun X. J. *et al.* Improved performance of GaN metal-semiconductor-metal ultraviolet detectors by depositing SiO2 nanoparticles on a GaN surface Appl. Phys. Lett. 98(12), 121117 (2011).

[b12] Chen M. C., Sheu J. K., Lee M. L., Kao C. J. & Chi G. C. Planar GaN p-i-n photodiodes with n+-conductive channel formed by Si implantation. Appl. Phys. Lett. 88(20), 203508 (2006).

[b13] Butun B., Tut T., Ulker E., Yelboga T. & Ozbay E. High-performance visible-blind GaN-based p - i - n photodetectors. Appl. Phys. Lett. 92(3), 033507 (2008).

[b14] Wang X. *et al.* Study of gain and photoresponse characteristics for back-illuminated separate absorption and multiplication GaN avalanche photodiodes. Appl. Phys. Lett. 115(1), 013103 (2014).

[b15] Carrano J. C. *et al.* Very low dark current metal-semiconductor-metal ultraviolet photodetectors fabricated on single-crystal GaN epitaxial layers. Appl. Phys. Lett. 70(15), 1992–1994 (1997).

[b16] Walker D. *et al.* High-speed, low-noise metal-semiconductor-metal ultraviolet photodetectors based on GaN. Appl. Phys. Lett. 74(5), 762–764 (1999).

[b17] Li D. B. *et al.* Effect of asymmetric Schottky barrier on GaN-based metal-semiconductor-metal ultraviolet detector. Appl. Phys. Lett. 99(26), 261102 (2011).

[b18] Sze S. M. Physics of Semiconductor Devices, 2nd ed. Wiley, New York, 1981.

[b19] Katz O., Garber V., Meyler B., Bahir G. & Salzman J. Gain mechanism in GaN Schottky ultraviolet detectors. Appl. Phys. Lett. 79(10), 1417–1419 (2001).

